# Efficacy and safety of intravenous methadone for pain management in cardiac surgery: a systematic review and meta-analysis

**DOI:** 10.3389/fmed.2026.1785811

**Published:** 2026-08-10

**Authors:** Wei Li, Yu Ye, Jianqiong Zhou, Yixin Ji, Zhengqun Gong

**Affiliations:** 1 School of Basic Medical Sciences & School of Nursing, Chengdu University, Chengdu, China; 2 Anesthesia Surgery Center, Affiliated Hospital of Chengdu University, Chengdu, China; 3 Department of Pain Medicine, Clinical Medical College and Affiliated Hospital of Chengdu University, Chengdu, China

**Keywords:** analgesia, cardiac surgical procedures, methadone, opioid, postoperative pain

## Abstract

**Objectives:**

To evaluate the analgesic efficacy and safety of intraoperative intravenous methadone compared with conventional opioid-based analgesic regimens in adult patients undergoing cardiac surgery, using data derived from randomized controlled trials and retrospective cohort studies.

**Methods:**

A comprehensive retrieval of scholarly literature was implemented in Embase, MEDLINE, PubMed, Web of Science, and the Cochrane Library until November 28, 2025. Randomized controlled trials and retrospective cohort studies comparing intraoperative methadone with other opioid analgesics for pain management in patients undergoing cardiac surgical procedures were eligible for inclusion in this meta-analysis. Quality of included studies was independently evaluated by two reviewers, with randomized controlled trials assessed using the Cochrane Risk of Bias tool (version 2.0) and cohort studies appraised using the Newcastle-Ottawa Scale. The primary outcome was postoperative pain intensity at 24 h. Secondary outcomes included postoperative 24-h opioid consumption, time to first rescue morphine administration, time to extubation, ICU length of stay, hospital length of stay, and reported adverse outcomes, including postoperative nausea, vomiting, and postoperative reintubation.

**Results:**

Eight studies, including 4 randomized controlled trials and 4 retrospective cohort studies, involving 10,203 patients were included. Compared with conventional opioid analgesics, intraoperative methadone was associated with lower postoperative 24-h pain intensity (SMD, −0.44; 95% CI − 0.71 to −0.17; *p* = 0.001, I^2^ = 77%). Methadone was also associated with lower postoperative 24-h opioid consumption in the primary analysis (SMD, −0.72; 95% CI, −1.35 to −0.23; *p* = 0.02; I^2^ = 97%), No statistically significant differences were observed for overall time to first rescue morphine administration, time to extubation, ICU length of stay, or hospital length of stay. In randomized controlled trials, methadone was not associated with significant differences in postoperative nausea or vomiting, whereas reintubation was less frequent in the methadone group (RR, 0.75; 95% CI, 0.58 to 0.96; *p* = 0.02). In retrospective cohort studies, postoperative nausea and vomiting were slightly less frequent with methadone (RR, 0.96; 95% CI, 0.93 to 1.00; *p* = 0.04), but the magnitude of this association was small.

**Conclusion:**

In adult patients undergoing cardiac surgery, intraoperative intravenous methadone may be associated with lower pain intensity and reduced opioid consumption during the first 24 h after surgery. However, substantial heterogeneity, inconsistent findings between randomized and retrospective studies, and incomplete reporting of key safety outcomes limit the certainty of the evidence. These findings should be interpreted cautiously, and further adequately powered randomized trials with standardized analgesic protocols and safety monitoring are needed.

**Systematic review registration:**

https://www.crd.york.ac.uk/prospero/display_record.php?ID=CRD420251237093, identifier (CRD420251237093).

## 1 Introduction

For patients undergoing cardiac surgical procedures, opioid pain medications still stand as the foundational component of perioperative pain control, because of their potent *μ*-opioid receptor-mediated analgesic effects and their ability to blunt sympathetic nervous system activation induced by surgical stress (1). Opioids such as fentanyl, morphine, hydromorphone, and sufentanil are routinely administered during and after cardiac surgery. A contemporary large-cohort retrospective study utilizing a multicenter perioperative database in the United States reported that among Adult patients undergoing coronary artery bypass grafting or cardiac valvular procedures, more than 99% received at least one opioid intraoperatively. Fentanyl was the most frequently used agent, administered in approximately 86% of cases, while morphine, hydromorphone, and sufentanil were also commonly employed (2).

However, high-dose opioid administration has been associated with opioid tolerance, physical dependence, postoperative nausea and vomiting, gastrointestinal dysfunction, and delayed functional recovery (3–5). In addition, opioid-related respiratory depression, prolonged time to endotracheal tube removal, and Opioid-related respiratory depression, delayed extubation, and prolonged intensive care unit stay are particularly relevant in cardiac surgery because they may adversely affect postoperative recovery and increase health care resource utilization (6). Therefore, opioid-sparing or opioid-optimizing strategies have emerged as a key component of modern perioperative care pathways.

Methadone, a synthetic opioid, is characterized by a prolonged and variable half-life of elimination as well as antagonistic activity at N-methyl-D-aspartate (NMDA) receptors (7). These pharmacologic characteristics imply that a single IV methadone dose given during surgery may provide sustained postoperative analgesia during the period of greatest pain intensity, potentially alleviating the need to administer short-acting opioids repeatedly. Accordingly, methadone has emerged as a proposed component of multimodal pain management strategies for cardiac surgery (8). However, its adoption remains limited, largely because of persistent safety concerns, including QT interval prolongation, arrhythmias, and delayed respiratory depression (9).

Clinical evidence concerning methadone’s analgesic performance in cardiac surgical procedures remains inconsistent. Randomized trials suggest that administering intravenous methadone during surgery may reduce pain experienced postoperatively and reduce overall opioid use (10), whereas observational studies have reported more modest and nonsignificant improvements in pain outcomes (11). Heterogeneity across research design, dosage strategies, comparator opioids, and assessment variables further limits the interpretability of existing data.

Accordingly, we carried out a systematic review and meta-analysis designed to evaluate the effectiveness and safety profile of intravenous methadone for perioperative analgesia in adult individuals undergoing cardiac surgical procedures, with a focus on postoperative pain severity, opioidutilization, and opioid-associated adverse reactions. This study aims to provide a quantitative synthesis of existing evidence to inform perioperative analgesic decision-making in cardiac surgical practice.

## 2 Methods

This systematic review and meta-analysis was registered in PROSPERO before study selection was completed (2025 CRD420251237093), the study was conducted and reported in accordance with the Preferred Reporting Items for Systematic Reviews and Meta-Analyses (PRISMA) 2020 statement. The methodological conduct of the review was also informed by the AMSTAR-2 checklist (12). Given that this research relied solely on data published previously and did not incorporate patient-specific individual information, ethical clearance and informed consent were deemed unnecessary.

### 2.1 Strategy for literature searching

A comprehensive literature search was conducted in PubMed, Web of Science, Embase, MEDLINE, and the Cochrane Library from database inception to November 28, 2025, to identify eligible studies comparing intraoperative intravenous methadone with other opioid analgesics, including morphine, fentanyl, sufentanil, or hydromorphone, for postoperative pain management in adult patients undergoing cardiac surgery. Articles published online ahead of print before the final search date were considered eligible for inclusion if they met the prespecified eligibility criteria. The search strategy integrates the following related terms: “methadone,” “analgesia,” “cardiac surgery,” “cardiac surgical procedure,” “cardiothoracic surgery, “and “heart surgery.” The search was not limited by language, time of publication, or cardiac surgical procedure. The complete search strategies are provided in Supplementary Table 1.

### 2.2 Study selection

Search results were imported into EndNote software, and duplicate records were removed. Two reviewers independently screened titles and abstracts for eligibility. When the title or abstract provided insufficient information to determine eligibility, the full text was reviewed. Disagreements were resolved through discussion or, when necessary, consultation with a third independent reviewer.

### 2.3 Study inclusion and exclusion criteria

Studies met the eligibility criteria for inclusion only if they satisfied all subsequent criteria: (i) randomized controlled trials (RCTs) or observational studies that enrolled adult patients (≥18 years); (ii) studies in which intravenous methadone was administered intraoperatively for perioperative pain management and compared with conventional opioid analgesics (e.g., morphine, fentanyl, sufentanil, or hydromorphone); (iii) studies reporting postoperative pain-related outcomes and/or opioid-related adverse events, including pain intensity assessed using validated pain scales and/or postoperative opioid consumption; (iv) studies providing sufficient data to allow extraction or calculation of effect estimates for outcomes of relevance.

The exclusion requirements were specified as below: (i) methadone administered via nonintravenous routes;(ii) non–cardiac surgical populations;(iii) insufficient outcome data for quantitative synthesis;(iv) case reports, case series, conference abstracts without full-text availability, reviews, meta-analyses, letters, duplicate publications or animal studies.

### 2.4 Defining study outcome metrics

The primary outcome was postoperative pain intensity at 24 h after surgery, assessed using validated pain scoring instruments. Secondary outcomes included postoperative 24-h opioid consumption, time to first rescue morphine administration, time to extubation, ICU length of stay, hospital length of stay, and reported adverse outcomes, including postoperative nausea, vomiting, and postoperative reintubation. Additional postoperative pain scores at other time points, including pain at rest and during coughing at 48 and 72 h and average pain scores within 24 to 48 h, were extracted and analyzed when available.

### 2.5 Extracting study data

Extracting study data was independently executed by two review authors via a standardized, pre-established electronic data collection tool. Any inconsistencies identified were addressed via group discussion and, if required, consultation involving a third independent researcher. The abstracted data encompassed first author names, publication year, and study type, country, type of surgery, exclusion criteria, intervention characteristics, sample size, methadone dosage, route of administration, and postoperative outcomes.

For quantitative synthesis, continuous or ordinal outcome measures were extracted as measured means alongside their respective standard deviations (SDs). These outcomes included postoperative pain scores at 24 h; pain scores at rest and during coughing at 48 and 72 h; average pain scores within 24 to 48 h postoperatively; postoperative opioid analgesic utilization within 24 h; time to first rescue morphine administration; time to extubation; hospital stay duration, and ICU stay duration. To maintain consistency across included studies, all opioid pain medication doses were standardized to oral morphine equivalents (OME) by employing a pre-validated opioid conversion scale (13), the total 24-h OME was calculated using the following formula:


$$\mathrm{OM}E_{24h}=\sum_{i=1}^{n}({\text{Dose}}_{i}\times {\text{Conversion factor}}_{i})$$

The following conversion factors were used: 1 mg of oral morphine was equivalent to 1 mg of OME, and 1 mg of intravenous morphine was equivalent to 3 mg of OME. For studies that reported postoperative opioid consumption as actual intravenous morphine doses, the reported values were multiplied by 3 to convert them to OMEs. Values from studies that directly reported postoperative opioid consumption as OMEs were extracted without additional conversion.

Dichotomous outcomes, including postoperative nausea, vomiting, and reintubation were collected as event counts. For studies reporting continuous outcomes a presented as medians with interquartile ranges (IQRs) or medians with full data ranges, as an alternative to means and standard deviations (SDs), the data were transformed into derived means and their SDs based on the statistical methods proposed by Wan et al. (14) and Luo et al. (15), time-related outcomes were converted to a common unit for each outcome before pooling.

### 2.6 Evaluating bias-related risk

For randomized controlled trials, risk of bias was independently appraised by two reviewers using the Cochrane Risk of Bias tool, version 2 (RoB 2.0) (16). The following assessment areas underwent evaluation: the process of random sequence generation, allocation concealment, deviations from the originally planned interventions, masking of outcome measurement, outcome data completeness, and selective outcome reporting. Every assessment domain was judged as having a “low risk of bias,” “high risk of bias,” or “some concerns.” For observational studies, methodological completeness was appraised via the Newcastle–Ottawa Scale (NOS) (17), which evaluates three domains: study group selection, comparability between groups, and assessment of outcomes. Based on total NOS scores, included studies were classified as demonstrating high (7–9 points), moderate (4–6 points), or low (0–3 points) levels of methodological rigor. In addition, the certainty of evidence for each outcome was assessed using the Grading of Recommendations Assessment, Development and Evaluation (GRADE) approach (18). Evidence certainty was rated as high, moderate, low, or very low according to risk of bias, inconsistency, indirectness, imprecision, and publication bias. Disagreements during risk-of-bias or certainty assessments were resolved by discussion.

### 2.7 Statistical analysis

For continuous outcomes, including pooled effect estimates were calculated as mean differences (MDs) alongside their corresponding 95% confidence intervals (CIs). Regarding 24-h postoperative morphine utilization volume and pain scores, Effect size estimates were pooled into standardized mean differences (SMDs) together with 95% confidence intervals (CIs) using an inverse-variance method, due to the employment of divergent measurement tools across included studies. For dichotomous outcomes, including postoperative nausea, vomiting, and reintubation, Summary effect size estimates were computed as risk ratios (RRs) with corresponding 95% CIs (19). Assessment of statistical heterogeneity across included studies was evaluated via the Cochrane Q test and quantified using the I^2^ heterogeneity statistic. A fixed-effect model was used when heterogeneity was low (I^2^ ≤ 50% and *p* ≥ 0.10). A random-effects model was used when substantial heterogeneity was present (I^2^ > 50% or *p* < 0.10) (19). Leave-one-out sensitivity assessments were conducted for the primary outcome and for outcomes with substantial heterogeneity, in these analyses, each study was sequentially removed to assess the robustness of the pooled effect estimates. Small-study effects for the primary outcome were assessed qualitatively by visual inspection of funnel plot asymmetry. Because fewer than 10 studies were included, formal statistical tests for publication bias were not performed. All analyses were conducted using Review Manager 5.4. All statistical tests were 2-sided, and *p* < 0.05 was considered statistically significant.

## 3 Result

### 3.1 Selection and characteristics of included studies

**Figure 1:**
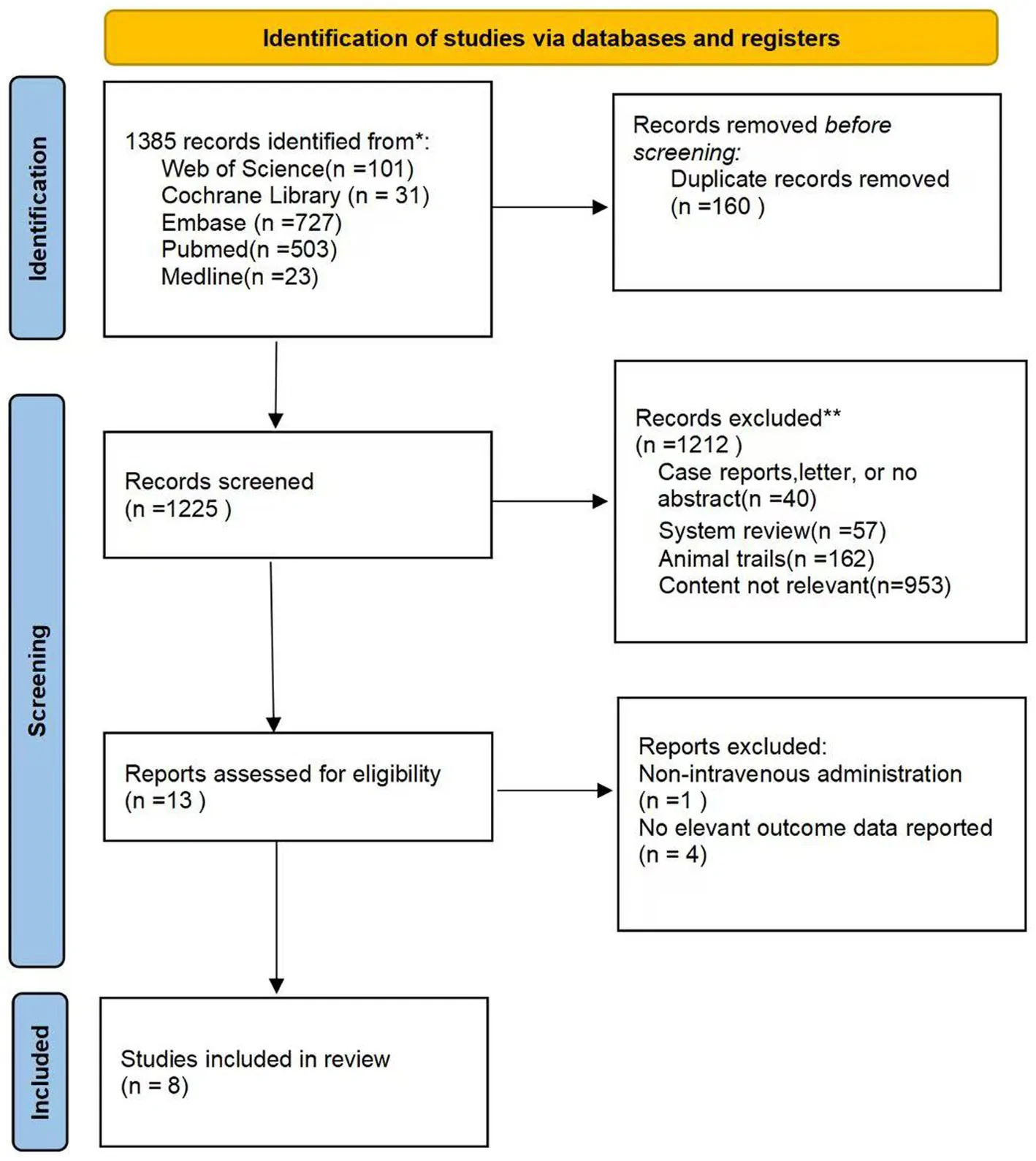
PRISMA flow diagram.

Database searches identified 1,385 potential records, of which 160 duplicates were removed. After title and abstract screening, 1,212 records were excluded because they did not meet the inclusion criteria. The remaining 13 full-text articles were assessed for eligibility, and 8 studies were ultimately included in the meta-analysis (10, 11, 20–25) in the meta-analysis. The selection workflow is illustrated in Figure 1.

Eight eligible studies encompassing 10,203 patients were incorporated into the meta-analysis, comprising 4 randomized controlled trials and 4 retrospective cohort studies. Five studies were conducted in the United States, 2 in Brazil, and 1 in China. Except for the study by Udelsmann et al. (22), all included studies specified the types of cardiac surgery in their eligibility criteria. The key attributes of the studies incorporated in this meta-analysis are systematically compiled in Supplementary Table 2.

### 3.2 Description of the quality of included trials

**Figure 2:**
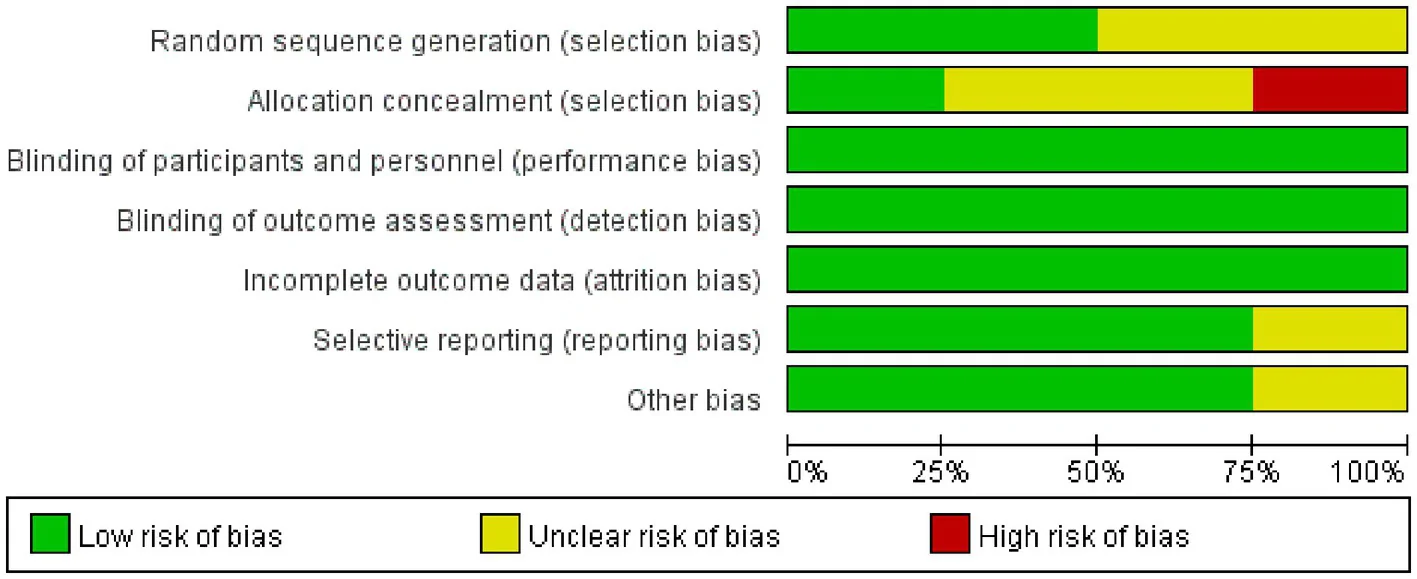
Risk-of-bias assessment for randomized controlled trials.

Risk of bias was assessed for all enrolled studies, and observational studies exhibited generally high methodological rigor, all achieving a Newcastle–Ottawa Scale (NOS) score of ≥7 (Supplementary Table 3), All randomized controlled trials reported randomization; however, 2 trials did not provide sufficient details regarding sequence generation or allocation concealment. The domains of blinding and completeness of outcome data were consistently assessed as low risk of bias (Figure 2).

### 3.3 Postoperative 24-h pain intensity

**Figure 3:**
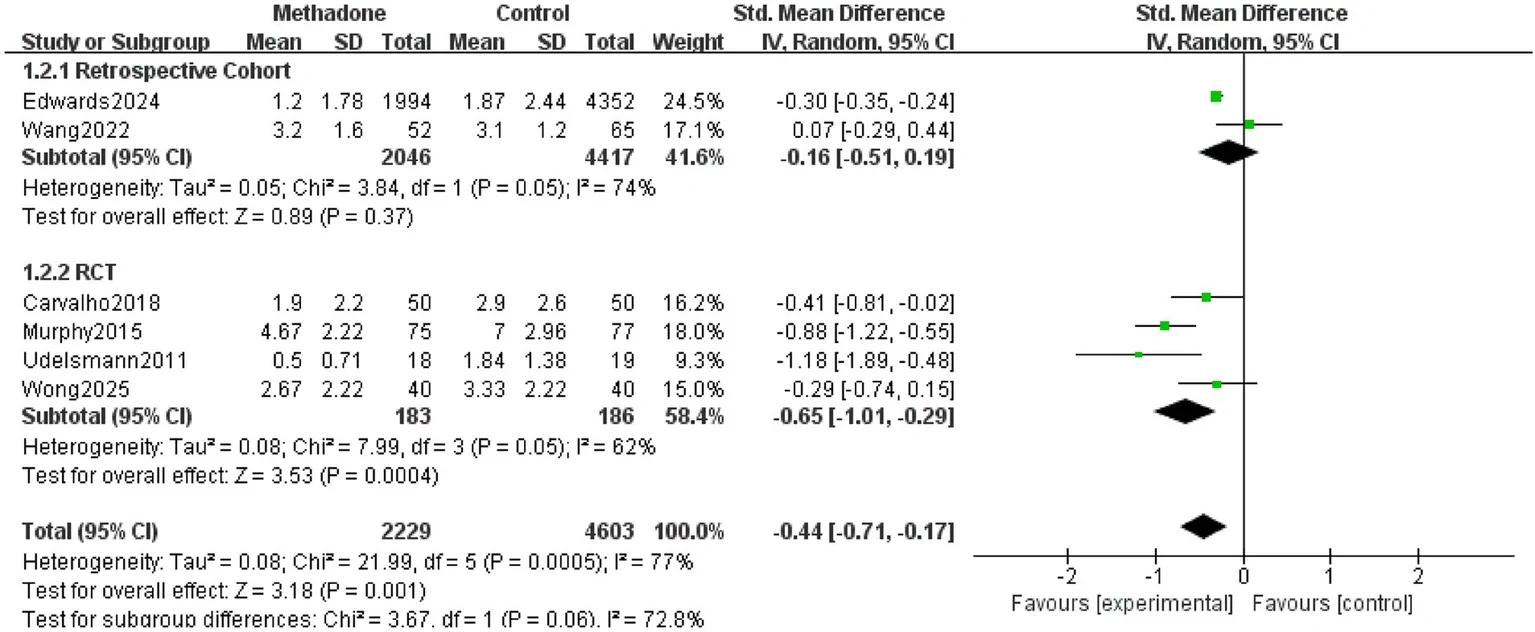
Forest plot comparing postoperative 24-h pain intensity between methadone and control opioids.

Pain intensity was assessed using different validated scales, including the 0- to 10-point Numerical Rating Scale (NRS) (20, 21, 24) or Numeric Pain Rating Scale (NPRS) (10), and Visual Analog Scale (VAS) (22). Therefore, standardized mean differences (SMDs) were used for pooled analyses. Compared with conventional opioid analgesics, methadone administration was linked to markedly lower 24-h postoperative pain intensity scores (SMD, −0.44; 95% CI, −0.71 to −0.17; *p* = 0.001, I^2^ = 77%). In subgroup analyses by study design, randomized controlled trials displayed a pronounced decrease in postoperative 24-h pain scores in the methadone group (SMD, −0.65; 95% CI, −1.01 to −0.29; *p* = 0.0004; I^2^ = 62%). In opposition, retrospective cohort analyses lacked evidence of a significant statistical difference between the study groups (SMD, −0.16; 95% CI, −0.51 to 0.19; *p* = 0.37; I^2^ = 74%) (Figure 3).

To further explore potential sources of heterogeneity, a leave-one-out sensitivity analysis was conducted. After exclusion of Murphy et al. (10, 26), the association between methadone and lower postoperative 24-h pain scores remained statistically significant (SMD, −0.32; 95% CI, −0.56 to −0.08; *p* = 0.008), and between-study heterogeneity decreased from I^2^ = 77% to I^2^ = 61% (Supplementary Figure 1). These findings suggest that Murphy et al. (10, 26) may have contributed to the observed heterogeneity. One possible explanation is that Murphy et al. (10, 26) used a higher methadone dose than most other included studies, which may have produced a larger analgesic effect. However, residual heterogeneity after exclusion of this study indicates that other study-level factors may also have contributed, including differences in comparator opioids, surgical procedures, cardiopulmonary bypass use, perioperative analgesic protocols, and pain assessment methods.

Visual inspection of the funnel plot for the primary outcome did not reveal clear evidence of asymmetry (Supplementary Figure 2). Additional leave-one-out sensitivity analyses showed that sequential exclusion of individual studies did not materially change the direction or statistical significance of the pooled effect estimate (Supplementary Table 4).

### 3.4 Postoperative pain intensity at additional time points

Five studies reported pain scores across varying postoperative time points and under different conditions, including resting pain at the 48 and 72 h, pain during coughing at the 48 and 72 h, pain during coughing at 48 and 72 h, and average pain intensity during the 24- to 48-h postoperative period. For pain at rest, no statistically significant difference was observed between the methadone and control groups at 48 h postoperatively (MD, −0.80; 95% CI, −1.77 to 0.18; *p* = 0.11, I^2^ = 71%) (Supplementary Figure 3A). At 72 h, methadone was associated with lower pain scores compared with control opioids (MD, −0.48; 95% CI, −0.94 to −0.03; *p* = 0.04, I^2^ = 28%) (Supplementary Figure 3B). For pain during coughing, methadone was associated with lower pain scores at 48 h (MD, −1.47; 95% CI, −2.45 to −0.50; *p* = 0.003; I^2^ = 59%) (Supplementary Figures 3C,D)and 72 h (MD, −1.10; 95% CI, −1.78 to −0.43; *p* = 0.001; I^2^ = 0%). For average pain scores within 24 to 48 h postoperatively, no statistically difference was observed between groups (SMD, 0.19; 95% CI, −0.01 to 0.39; *p* = 0.06, I^2^ = 0%) (Supplementary Figure 3E).

### 3.5 Postoperative 24-h opioid consumption

**Figure 4:**
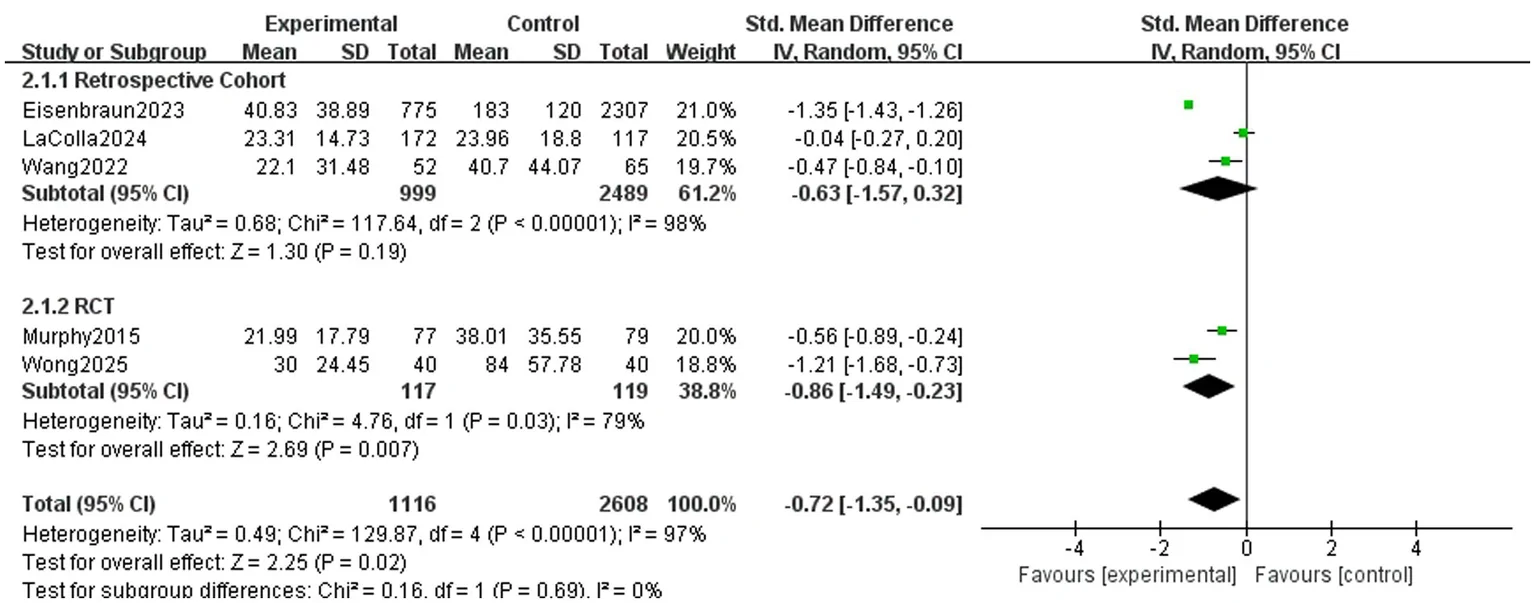
Forest plot comparing postoperative 24-h opioid consumption between methadone and control opioids.

2 randomized controlled trials and 3 retrospective cohort studies reported postoperative 24-h opioid consumption. Overall, intraoperative methadone was associated with lower postoperative 24-h opioid consumption compared with control opioids (SMD, −0.72; 95% CI, −1.35 to −0.23; *p* = 0.02, I^2^ = 97%). In subgroup analyses by study design, randomized controlled trials showed a significant reduction in postoperative 24-h opioid consumption in the methadone group (SMD, −0.86; 95% CI, −1.49 to −0.23; *p* = 0.007, I^2^ = 79%), whereas retrospective cohort studies showed no statistically significant difference between groups (SMD, −0.63; 95% CI, −1.57 to 0.32; *p* = 0.19, I^2^ = 98%) (Figure 4).

To further explore potential sources of heterogeneity, a leave-one-out sensitivity analysis was conducted. After exclusion of the large-sample study by Eisenbraun et al. (25), the pooled effect remained statistically significant (SMD, −0.54; 95% CI, −0.99 to −0.09; *p* = 0.02). However, between-study heterogeneity remained high, although it decreased from I^2^ = 97% to I^2^ = 86% (Supplementary Figure 4). This residual heterogeneity may be related to differences in surgical procedures, cardiopulmonary bypass use, comparator opioids, perioperative analgesic protocols, and opioid dose reporting methods.

### 3.6 Time to first rescue morphine

**Figure 5:**
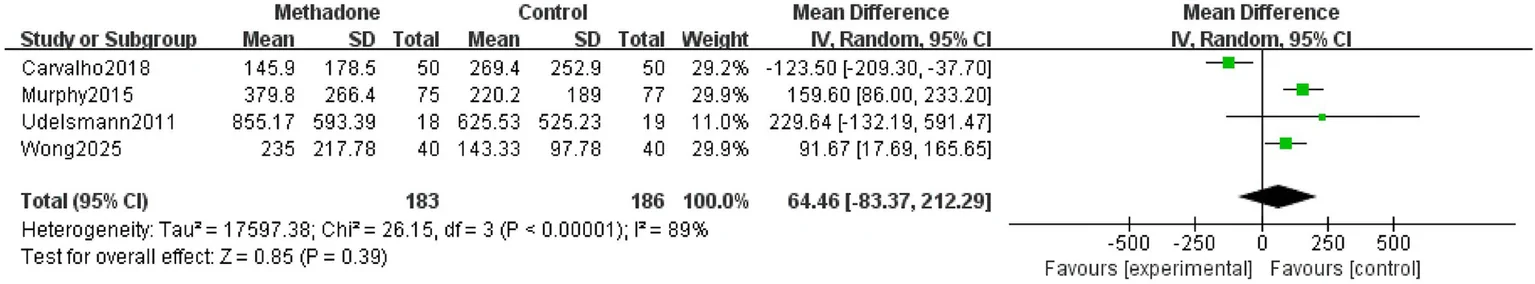
Forest plot comparing time to first rescue morphine administration between methadone and control opioids.

Four randomized controlled trials reported time to first rescue morphine administration. In the pooled analysis, no statistically significant difference was observed between the methadone and control groups (MD, 64.46; 95% CI, −83.37 to 212.29; *p* = 0.39; I^2^ = 89%) (Figure 5). To explore the potential source of heterogeneity, a leave-one-out sensitivity analysis was performed. After exclusion of Carvalho et al. (21), methadone was associated with a longer time to first rescue morphine administration compared with control opioids (MD, 127.92; 95% CI, 76.28 to 179.57; *p* < 0.001), and heterogeneity decreased from I^2^ = 89% to I^2^ = 0% (Supplementary Figure 5). This finding suggests that Carvalho et al. (21) may have been a major contributor to the heterogeneity observed in the overall analysis. A possible explanation is that Carvalho et al. (21) differed from the other included studies in cardiopulmonary bypass use and showed an effect estimate in the opposite direction. However, because only 3 studies remained after exclusion, this sensitivity analysis should be considered exploratory.

### 3.7 Time to extubation

**Figure 6:**
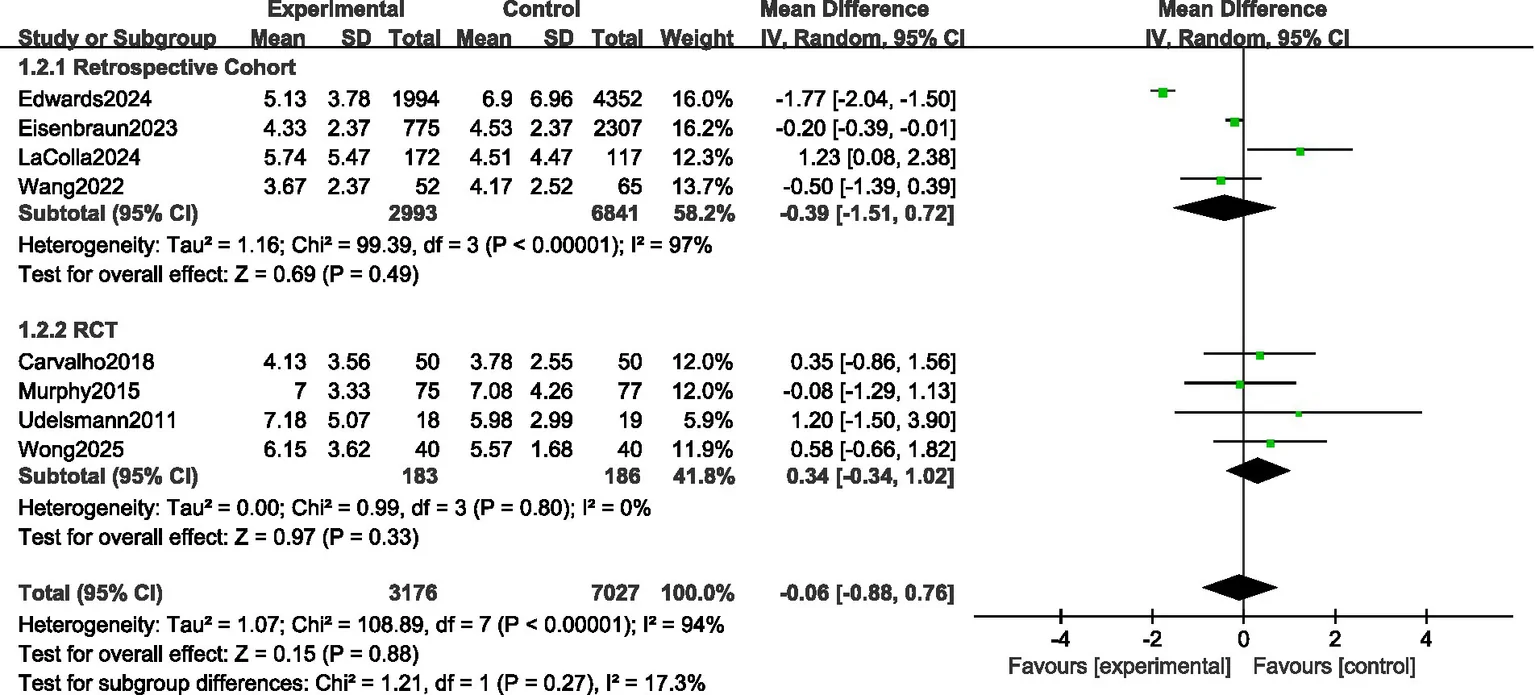
Forest plots comparing postoperative time to extubation between methadone and control opioids.

Four randomized controlled trials and four retrospective cohort studies reported postoperative time to extubation, In the overall analysis, no statistically significant difference was observed between the methadone and control groups (MD, −0.06; 95% CI, −0.88 to 0.76; *p* = 0.88, I^2^ = 94%). Subgroup analyses showed no statistically significant difference in retrospective cohort studies (MD, −0.39; 95% CI, −1.51 to 0.72; *p* = 0.49; I^2^ = 97%) or randomized controlled trials (MD, 0.34; 95% CI, −0.34 to 1.02; *p* = 0.33; I^2^ = 0%) (Figure 6). After exclusion of the large-sample study by Edwards et al. (11), heterogeneity in the retrospective cohort subgroup decreased from I^2^ = 97% to I^2^ = 36%, while the pooled effect estimate remained statistically nonsignificant. This finding suggests that Edwards et al. (11) may have influenced the degree of heterogeneity, but the persistence of residual heterogeneity indicates that additional study-level factors may also have contributed, such as differences in surgical populations, perioperative ventilation and extubation protocols, cardiopulmonary bypass use, institutional enhanced recovery pathways, and extubation criteria (Supplementary Figure 6).

### 3.8 Hospital length of stay

**Figure 7:**
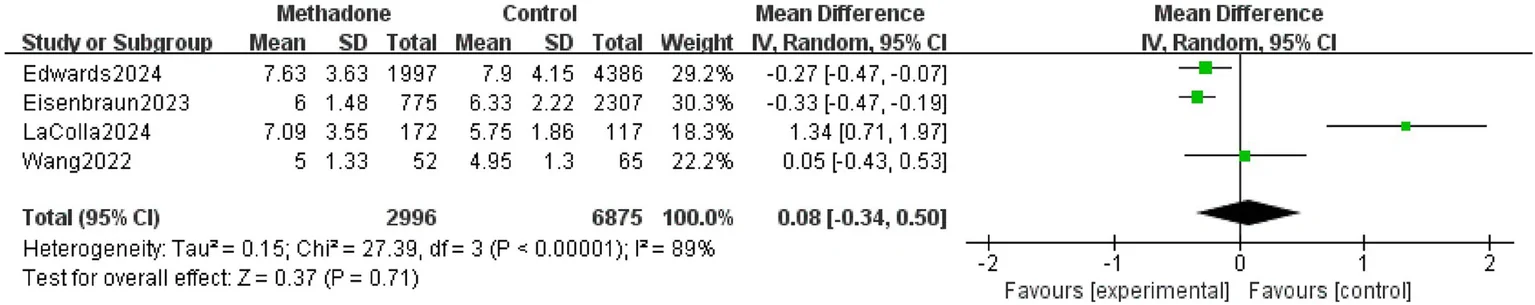
Forest plot comparing hospital length of stay between methadone and control opioids.

Four retrospective cohort studies compared postoperative hospital length of stay between patients receiving methadone and those receiving conventional opioid analgesics. No statistically significant difference was observed between groups (MD, 0.08; 95% CI, −0.34 to 0.50; *p* = 0.71, I^2^ = 89%) (Figure 7).

### 3.9 ICU length of stay

**Figure 8:**
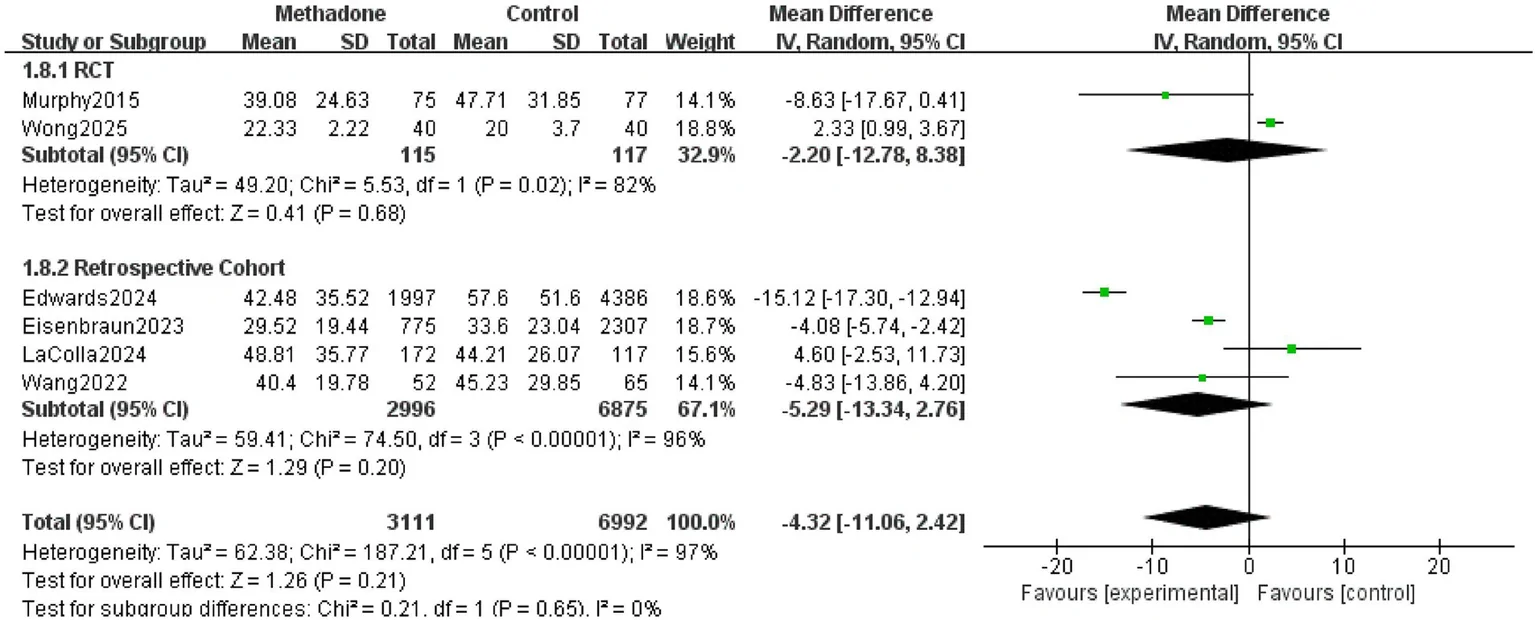
Forest plot comparing ICU length of stay between methadone and control opioids.

Two randomized controlled trials and four retrospective cohort studies reported postoperative ICU length of stay. In the overall analysis, no statistically meaningful discrepancy was detected between the methadone and control opioid groups (MD, −4.32; 95% CI, −11.06 to 2.42; *p* = 0.21, I^2^ = 97%). Subgroup analyses showed no evidence of shorter ICU stay with methadone in randomized controlled trials (MD, −2.20; 95% CI, −12.78 to 8.38; *p* = 0.68, I^2^ = 82%) or retrospective cohort studies (MD, −5.29; 95% CI, −13.34 to 2.76; *p* = 0.20, I^2^ = 96%) (Figure 8).

A leave-one-out sensitivity analysis was conducted to assess the robustness of the pooled estimate and explore potential sources of heterogeneity. Sequential exclusion of individual studies did not substantially reduce heterogeneity, and the pooled effect estimate remained statistically nonsignificant. These findings suggest that no single study fully accounted for the substantial heterogeneity observed for ICU length of stay; rather, the heterogeneity may be related to differences in surgical populations, cardiopulmonary bypass use, perioperative ICU management, institutional discharge criteria, and enhanced recovery protocols.

### 3.10 Impact on incidence of adverse outcomes

Four randomized controlled trials reported postoperative nausea and vomiting. Methadone was not associated with a statistically significant difference in postoperative nausea (RR 0.85; 95% CI, 0.66 to 1.08; *p* = 0.18; I^2^ = 0%) (Supplementary Figure 7A) or vomiting (RR, 0.99; 95% CI, 0.65 to 1.50; *p* = 0.96; I^2^ = 15%) (Supplementary Figure 7B). Postoperative reintubation was less frequent in the methadone group (RR, 0.75; 95% CI, 0.58 to 0.96; *p* = 0.02; I^2^ = 0%) (Supplementary Figure 7C). In retrospective cohort studies, postoperative nausea and vomiting were slightly less frequent in the methadone group than in the control group (RR, 0.96; 95% CI, 0.93 to 1.00; *p* = 0.04; I^2^ = 0%) (Supplementary Figure 7D). However, the effect estimate was close to the null value, and the clinical significance of this finding is limited.

### 3.11 Certainty of evidence

The GRADE assessment rated the certainty of evidence as moderate for postoperative 24-h pain scores and postoperative nausea and vomiting, and low for all other outcomes. Evidence certainty was downgraded primarily because of substantial inconsistency, imprecision related to wide confidence intervals or limited numbers of contributing studies, and, in some cases, study design limitations because evidence was derived partly or predominantly from observational studies. Although methadone was associated with lower early postoperative pain scores and opioid consumption, these findings should be interpreted cautiously because of substantial heterogeneity and potential residual confounding. The observed reduction in postoperative nausea and vomiting in retrospective cohort studies was small in magnitude and close to the null value; therefore, this finding should be considered exploratory and of limited clinical significance (Supplementary Table 5).

## 4 Discussion

The present systematic review and meta-analysis assessed the analgesic efficacy and safety of intraoperative intravenous methadone compared with conventional opioid-based analgesic regimens in adult patients undergoing cardiac surgery. The pooled results suggest that methadone was associated with lower postoperative pain intensity and reduced opioid consumption within the first 24 h after surgery. These associations appeared more pronounced in randomized controlled trials than in retrospective cohort studies. However, no consistent benefit was observed for time to first rescue morphine administration, time to extubation, ICU length of stay, hospital length of stay, or most reported short-term adverse events.

These findings should be interpreted cautiously because substantial clinical and methodological heterogeneity was observed. Although subgroup analyses by study design were performed, heterogeneity remained considerable for postoperative 24-h pain scores and postoperative 24-h opioid consumption. This suggests that study design alone did not fully explain the between-study variability. Potential contributors include differences in surgical procedures, cardiopulmonary bypass use, comparator opioids, intraoperative methadone dose, perioperative analgesic protocols, postoperative pain assessment methods, and opioid dose reporting or conversion methods.

Sensitivity analyses provided additional insight into the potential sources of heterogeneity. For postoperative 24-h pain scores, exclusion of Murphy et al. (10, 26) reduced heterogeneity from I^2^ = 77% to I^2^ = 61%, while the association between methadone and lower pain scores remained statistically significant. This suggests that Murphy et al. (10, 26) may have contributed to the observed heterogeneity. One possible explanation is that this trial used a higher methadone dose than most other included studies, which may have produced a larger analgesic effect. However, this effect cannot be separated from other study-level differences, including use of fentanyl as the comparator opioid, inclusion of mixed cardiac surgical procedures, and cardiopulmonary bypass use.

Similarly, for postoperative 24-h opioid consumption, exclusion of the large-sample study by Eisenbraun et al. (25) reduced heterogeneity from I^2^ = 97% to I^2^ = 86%, but substantial residual heterogeneity remained. This indicates that Eisenbraun et al. (25) may have influenced the pooled estimate, but it was unlikely to be the sole source of heterogeneity. Remaining variability may reflect differences in surgical populations, cardiopulmonary bypass use, comparator opioid regimens, perioperative analgesic pathways, opioid dose reporting, and conversion to oral morphine equivalents.

Therefore, the pooled effect estimates for early postoperative pain and opioid consumption should be viewed as suggestive rather than definitive evidence of a uniform treatment effect across all cardiac surgical populations. The greater apparent benefit observed in randomized controlled trials compared with retrospective cohort studies may also reflect differences in confounding control. Retrospective studies are inherently more susceptible to confounding by indication, selection bias, and incomplete adjustment for baseline clinical differences. For example, patients receiving methadone may have differed from those receiving conventional opioids in surgical complexity, institutional analgesic protocols, clinician preference, preoperative opioid exposure, or expected postoperative pain burden. Although several observational studies attempted statistical adjustment, residual confounding cannot be excluded. Accordingly, the combined estimates derived from randomized and nonrandomized evidence should be interpreted with caution.

Our findings are broadly consistent with previous literature suggesting that intraoperative methadone may reduce acute postoperative pain and opioid requirements after major surgery, including cardiothoracic procedures, without a clear increase in commonly reported short-term adverse events (26). However, the magnitude and consistency of benefit in cardiac surgery remain uncertain because of the limited number of trials, differences in methadone dosing strategies, and heterogeneity in comparator analgesic regimens. The available evidence suggests that the analgesic benefit of methadone is most apparent in the early postoperative period, particularly within the first 24 h after surgery.

Accumulating evidence also suggests that the benefits of intraoperative methadone may be time limited. Longitudinal follow-up data from randomized trials comparing methadone with fentanyl in cardiac surgical populations indicate that reductions in pain severity and pain-related interference are most pronounced during the early postoperative period and may attenuate over time (27). Taken together, these findings suggest that intraoperative methadone may be most useful as part of an early postoperative analgesic strategy rather than as an intervention expected to modify long-term pain trajectories or functional recovery after cardiac surgery.

The safety findings also require cautious interpretation. In the included studies, methadone was not associated with a consistent increase in postoperative nausea, vomiting, or reintubation. However, the available safety data were limited and incompletely reported. Key methadone-related safety outcomes, including QT interval prolongation, arrhythmia, respiratory depression, and depth of sedation, were not consistently assessed across studies. Therefore, the absence of a statistically significant increase in reported adverse events should not be interpreted as definitive evidence of safety. Future trials should include standardized electrocardiographic monitoring, respiratory safety assessment, sedation scoring, and longer-term follow-up.

Overall, the current evidence suggests that intraoperative intravenous methadone may reduce early postoperative pain intensity and opioid consumption in adult cardiac surgery patients. Nevertheless, because the certainty of evidence is limited by substantial heterogeneity, residual confounding in retrospective studies, and incomplete safety reporting, these findings should be interpreted cautiously. Further adequately powered randomized clinical trials using standardized methadone dosing, comparator opioid regimens, perioperative analgesic protocols, opioid conversion methods, and safety monitoring are needed to clarify the efficacy and safety of methadone in cardiac surgical populations.

## 5 Study limitations

This meta-analysis has several limitations. First, the inclusion of both randomized controlled trials and retrospective cohort studies introduced clinical and methodological heterogeneity. Although randomized trials provide stronger internal validity, retrospective studies are more susceptible to confounding by indication, selection bias, and incomplete adjustment for baseline clinical differences. Second, substantial statistical heterogeneity was observed for several outcomes, including postoperative 24-h pain scores, postoperative 24-h opioid consumption, ICU length of stay, and hospital length of stay. This heterogeneity may be attributable to differences in surgical procedures, cardiopulmonary bypass use, comparator opioids, methadone dosing, timing of administration, perioperative analgesic protocols, pain assessment methods, and opioid dose reporting or conversion methods. Although subgroup and sensitivity analyses were conducted, residual heterogeneity remained for several outcomes. Third, the certainty of evidence was low for most outcomes. Evidence was downgraded mainly because of inconsistency, imprecision, limited numbers of contributing studies, and study design limitations. Therefore, the observed associations between methadone and reduced early postoperative pain or opioid consumption should be considered suggestive rather than definitive. Fourth, safety data were incompletely reported. Most studies reported only selected short-term adverse events, such as postoperative nausea, vomiting, and reintubation. Key methadone-related safety outcomes, including QT interval prolongation, arrhythmia, respiratory depression, sedation depth, and electrocardiographic monitoring, were not consistently assessed. Finally, the majority of included studies focused on short-term postoperative outcomes. Only a single study reported longer-term endpoints such as 30-day mortality and readmission, no notable differences were identified across the treatment groups (23). The limited availability of long-term data precludes a comprehensive assessment of methadone’s effects on chronic pain development, opioid dependence, functional recovery, and long-term safety.

## 6 Conclusion

In adult patients undergoing cardiac surgery, intraoperative intravenous methadone may be associated with lower pain intensity and reduced opioid consumption during the first 24 h after surgery. No consistent differences were observed in perioperative recovery outcomes or most reported short-term adverse events. However, substantial heterogeneity, residual confounding in retrospective studies, and incomplete reporting of key safety outcomes limit the certainty of the evidence. These findings should be interpreted cautiously, and further large, rigorously designed randomized clinical trials with standardized dosing strategies, analgesic protocols, safety monitoring, and extended follow-up are needed.

## Data Availability

All data analyzed in this study were derived from previously published studies. The datasets supporting the conclusions of this article are included within the article and its Supplementary material.

## References

[1] Stein C. Opioid receptors. Annu Rev Med. (2016) 67:433–51. doi: 10.1146/annurev-med-062613-093100, 26332001

[2] Fisher C Janda AM Zhao X Deng Y Bardia A Yanez ND . Opioid dose variation in cardiac surgery: a multicenter study of practice. Anesth Analg. (2025) 140:1016–27. doi: 10.1213/ANE.0000000000007128, 39167548 PMC11842693

[3] Urman RD Khanna AK Bergese SD Deng Y Bardia A Yanez ND . Postoperative opioid administration characteristics associated with opioid-induced respiratory depression: results from the PRODIGY trial. J Clin Anesth. (2021) 70:110167. doi: 10.1016/j.jclinane.2021.110167 33493688

[4] Gan TJ Belani KG Bergese S Chung F Diemunsch P Habib AS . Fourth consensus guidelines for the management of postoperative nausea and vomiting. Anesth Analg. (2020) 131:411–448. doi: 10.1213/ANE.0000000000004833, 32467512

[5] Grant MC Chappell D Gan TJ Manning MW Miller TE Brodt JL . Pain management and opioid stewardship in adult cardiac surgery: joint consensus report of the PeriOperative quality initiative and the enhanced recovery after surgery cardiac society. J Thorac Cardiovasc Surg. (2023) 166:1695–1706.e2. doi: 10.1016/j.jtcvs.2023.01.020 36868931

[6] Ochroch J Usman A Kiefer J Pulton D Shah R Grosh T . Reducing opioid use in patients undergoing cardiac surgery—preoperative, intraoperative, and critical care strategies. J Cardiothorac Vasc Anesth. (2021) 35:2155–65. doi: 10.1053/j.jvca.2020.09.103, 33069556 PMC8285065

[7] Robinson KM Eum S Desta Z Tyndale RF Gaedigk A Crist RC . Clinical pharmacogenetics implementation consortium (CPIC). CPIC guideline for CYP2B6 genotype and methadone therapy. Clin Pharmacol Ther. (2024) 116:932–8. doi: 10.1002/cpt.3338 38863207 PMC11587193

[8] Fernandes RM Pontes JPJ Rezende Borges CE de Brito Neto DR Pereira A d J Carvalho VP . Multimodal analgesia strategies for cardiac surgery: a literature review. Heart. (2024) 5:349–64. doi: 10.3390/hearts5030025

[9] Alinejad S Kazemi T Zamani N Hoffman RS Mehrpour O. A systematic review of the cardiotoxicity of methadone. EXCLI J. (2014) 13:553–74. 10.17179/excli2015-553 PMC4747000 26869865

[10] Murphy GS Szokol JW Avram MJ Greenberg SB Marymont JH Shear T . Intraoperative methadone for the prevention of postoperative pain: a randomized, double-blinded clinical trial in cardiac surgical patients. Anesthesiology. (2015) 122:1112–22. doi: 10.1097/ALN.0000000000000633, 25837528

[11] Edwards JN Whitney MA Smith BB Fah MK Buckner Petty SA Durra OLE . The role of methadone in cardiac surgery for management of postoperative pain. BJA Open. (2024) 10:100270. doi: 10.1016/j.bjao.2024.100270, 38560623 PMC10978480

[12] Page MJ McKenzie JE Bossuyt PM Boutron I Hoffmann TC Mulrow CD . The PRISMA 2020 statement: an updated guideline for reporting systematic reviews. Int J Surg. (2021) 88:105906. doi: 10.1016/j.ijsu.2021.105906 33789826

[13] Nielsen S Degenhardt L Hoban B Gisev N. A synthesis of oral morphine equivalents for opioid utilisation studies. Pharmacoepidemiol Drug Saf. (2016) 25:733–7. doi: 10.1002/pds.3945, 26693665

[14] Wan X Wang W Liu J Tong T. Estimating the sample mean and standard deviation from the sample size, median, range and/or interquartile range. BMC Med Res Methodol. (2014) 14:135. doi: 10.1186/1471-2288-14-135, 25524443 PMC4383202

[15] Luo D Wan X Liu J Tong T. Optimally estimating the sample mean from the sample size, median, mid-range, and/or mid-quartile range. Stat Methods Med Res. (2018) 27:1785–805. doi: 10.1177/0962280216669183, 27683581

[16] Sterne JAC Savović J Page MJ Elbers RG Blencowe NS Boutron I . RoB 2: a revised tool for assessing risk of bias in randomised trials. BMJ. (2019) 366:l4898. doi: 10.1136/bmj.l4898, 31462531

[17] Wells G.A. Shea B. O’Connell D. Peterson J. Welch V. Losos M. . (2000) The Newcastle–Ottawa Scale (NOS) for Assessing the Quality of Nonrandomised Studies in meta-Analyses Ottawa Hospital Research Institute. Available online at: https://www.ohri.ca/programs/clinical_epidemiology/oxford.asp (Accessed November 20, 2025).

[18] Guyatt GH Oxman AD Akl EA Kunz R Vist GE Brozek J . GRADE guidelines: 1. Introduction—GRADE evidence profiles and summary of findings tables. J Clin Epidemiol. (2011) 64:383–94. doi: 10.1016/j.jclinepi.2010.04.026, 21195583

[19] Higgins JPT Thomas J. Chandler J. , Cochrane Handbook for Systematic Reviews of Interventions. Version 6.4. Cochrane; (2023). Available online at: https://training.cochrane.org/handbook (Accessed November 20, 2025).

[20] Wong HMK Lai VKW Chiu SLC Wong WT Wo SK Zuo JZ . Intravenous methadone for pain management in cardiac surgery: a randomised controlled trial with plasma concentration analysis. Anaesthesia. (2026) 81:51–61. doi: 10.1111/anae.16754, 40859435 PMC12747610

[21] Carvalho AC Sebold FJG Calegari PMG Oliveira BH Schuelter-Trevisol F. Comparison of postoperative analgesia with methadone versus morphine in cardiac surgery. Braz J Anesthesiol. (2018) 68:122–7. doi: 10.1016/j.bjan.2017.09.005, 29096877 PMC9391719

[22] Udelsmann A Maciel FG Servian DCM Reis E Azevedo TMD Melo MDS. Methadone and morphine during anesthesia induction for cardiac surgery: repercussions in postoperative analgesia and prevalence of nausea and vomiting. Rev Bras Anestesiol. (2011) 61:695–701. doi: 10.1016/S0034-7094(11)70078-2 22063370

[23] LaColla L Nanez MA Frabitore S Lavage DR Warraich N Luke C . Intravenous methadone versus intrathecal morphine as part of an enhanced recovery after cardiac surgery protocol on postoperative pain and outcomes: a retrospective cohort study. J Cardiothorac Vasc Anesth. (2024) 38:2314–23. doi: 10.1053/j.jvca.2024.06.032, 39043493

[24] Wang DJ Song P Nault KM. Impact of intraoperative methadone use on postoperative opioid requirements after cardiac surgery. Am J Health Syst Pharm. (2022) 79:636–42. doi: 10.1093/ajhp/zxab459, 34874991

[25] Eisenbraun A Schroeder D Schaff HV Brown MJ Stulak JM. Single-center retrospective comparison of opioid-based and multimodal analgesic regimens in adult cardiac surgery. J Cardiothorac Vasc Anesth. (2023) 37:1179–87. doi: 10.1053/j.jvca.2023.03.001, 37003853

[26] Lobova VA Roll JM Roll MLC. Intraoperative methadone use in cardiac surgery: a systematic review. Pain Med. (2021) 22:2827–34. doi: 10.1093/pm/pnab269, 34487175

[27] Murphy GS Avram MJ Greenberg SB Shear TD Deshur MA Dickerson D . Postoperative pain and analgesic requirements in the first year after intraoperative methadone for complex spine and cardiac surgery. Anesthesiology. (2020) 132:330–42. doi: 10.1097/ALN.0000000000003025, 31939849

