# Supplementary material for: Efficacy and safety of intravenous methadone for pain management in cardiac surgery: a systematic review and meta-analysis
Source: Front Med (Lausanne). 2026 Aug 10;13:1785811. doi: 10.3389/fmed.2026.1785811 (PMC13500751; doi:10.3389/fmed.2026.1785811)
Supplement: Supplementary file 1 [file Supplementary_file_1.pdf]

Supplementary Material

Supplementary Table 1. General Search Strategies for PubMed, Embase, Web of Science, Cochrane Library, and MEDLINE

Supplementary Table 2. Characteristics of the Included Studies

Supplementary Table 3. Newcastle–Ottawa Scale (NOS) scores for included observational studies

Supplementary Table 4: Sensitivity Analysis of Postoperative 24-hour pain score Between Methadone Group and Control Group

Supplementary Table 5. GRADE summary of findings for perioperative intravenous methadone versus conventional opioids in adult cardiac surgery

Supplementary Figure 1. Forest plot for the sensitivity analysis of postoperative 24-hour pain intensity after exclusion of Murphy et al.

Supplementary Figure 2. Funnel plot for postoperative pain score at 24 h

Supplementary Figure 3. Postoperative pain intensity at additional time points

Supplementary Figure 4. Forest plot for the sensitivity analysis of postoperative 24-hour opioid consumption after exclusion of Eisenbraun et al

Supplementary Figure 5. Forest plot for the sensitivity analysis of time to first rescue morphine administration after exclusion of Carvalho et al

Supplementary Figure 6. Forest plot for the sensitivity analysis of time to extubation after exclusion of Edwards et al

Supplementary 7: Forest plots comparing postoperative outcomes between methadone and control opioids(A)Nausea; (B)Vomiting; (C) Reintubation; (D) Nausea/Vomiting.

Supplementary Table 1. General Search Strategies for PubMed, Embase, Web of Science, Cochrane Library, and MEDLINE

Text S1 Search strategy
Database: Embase from inception to November 28, 2025
Search Strategy:
#1. 'methadone'/exp
#2. methadone,ab,kw OR dolophine,ab,kw
#3. #1 OR #2
#4. 'heart surgery'/exp OR 'cardiac surgery'/exp OR 'cardiovascular surgery'/exp OR 'coronary artery bypass grafting'/exp OR 'heart valve surgery'/exp OR 'cardiopulmonary bypass'/exp
#5. 'cardiac surgery',ab,kw OR 'cardiac surgical procedure',ab,kw OR 'cardiac surgical procedures',ab,kw OR 'cardiothoracic surgery',ab,kw OR 'heart surgery',ab,kw OR 'cardiovascular surgical procedure',ab,kw OR 'cardiovascular surgical procedures',ab,kw OR 'coronary artery bypass',ab,kw OR CABG,ab,kw OR 'coronary artery bypass graft',ab,kw OR 'coronary artery bypass grafting',ab,kw OR 'valve surgery',ab,kw OR 'valve replacement',ab,kw OR 'valve repair',ab,kw OR sternotomy,ab,kw OR 'cardiopulmonary bypass',ab,kw OR CPB,ab,kw
#6. #4 OR #5
#7. 'analgesia'/exp OR 'postoperative pain'/exp OR 'pain'/exp OR 'opioid analgesic agent'/exp OR 'morphine'/exp OR 'fentanyl'/exp OR 'sufentanil'/exp OR 'hydromorphone'/exp
#8. analgesia,ab,kw OR analgesic,ab,kw OR analgesics,ab,kw OR pain,ab,kw OR 'postoperative pain',ab,kw OR opioid,ab,kw OR opioids,ab,kw OR morphine,ab,kw OR fentanyl,ab,kw OR sufentanil,ab,kw OR hydromorphone,ab,kw
#9. #7 OR #8
#10. #3 AND #6 AND #9
Results: 727 records

Text S2 Search strategy
Database: Web of Science from inception to November 28, 2025
Search Strategy:
#1. TS=(methadone OR dolophine)
#2. TS=("cardiac surgery" OR "cardiac surgical procedure" OR "cardiac surgical procedures" OR "cardiothoracic surgery" OR "heart surgery" OR "cardiovascular surgical procedure" OR "cardiovascular surgical procedures" OR "coronary artery bypass" OR CABG OR "coronary artery bypass graft" OR "coronary artery bypass grafting" OR "valve surgery" OR "valve replacement" OR "valve repair" OR sternotomy OR "cardiopulmonary bypass" OR CPB)

#3. TS=(analgesia OR analgesic OR analgesics OR pain OR "postoperative pain" OR opioid OR opioids OR morphine OR fentanyl OR sufentanil OR hydromorphone)
#4. #1 AND #2 AND #3
Results: 101 records

Database: PubMed from inception to November 28, 2025
Search Strategy:
#1. "Methadone"[Mesh]
#2. (Phenadone OR Physeptone OR Phymet OR Dolophine OR Amidone OR "Methadone Hydrochloride" OR "Hydrochloride, Methadone" OR Biodone OR Metadol OR Metasedin OR Methaddict OR Methadose OR Methex OR Pinadone OR Symoron OR "intraoperative methadone" OR methadon)
#3. #1 OR #2
#4. "Cardiac Surgical Procedures"[Mesh]
#5. (Surgical Procedure, Cardiac OR Surgical Procedures, Cardiac OR Heart Surgical Procedure OR Heart Surgical Procedures OR Procedure, Heart Surgical OR Procedures, Heart Surgical OR Surgical Procedure, Heart OR Surgical Procedures, Heart OR Cardiac Surgical Procedure OR Procedures, Cardiac Surgical OR Procedure, Cardiac Surgical OR Cardiac Surgery OR Heart Surgery OR Cardiosurgery OR heart operation OR myocardial resection OR surgery, heart)
#6. #4 OR #5
#7. "Analgesics, Opioid"[Mesh]
#8. (Analgesic, Opioid OR Opioid Analgesics OR Opioids OR Full Opioid Agonists OR Agonists, Full Opioid OR Opioid Agonists, Full OR Opioid Full Agonists OR Agonists, Opioid Full OR Full Agonists, Opioid OR Opioid Mixed Agonist-Antagonists OR Agonist-Antagonists, Opioid Mixed OR Mixed Agonist-Antagonists, Opioid OR Partial Opioid Agonists OR Agonists, Partial Opioid OR Opioid Agonists, Partial OR Opioid Partial Agonists OR Agonists, Opioid Partial OR Partial Agonists, Opioid)
#9. #7 OR #8
#10. #3 AND #6 AND #9
Results: 503 records

Database: Cochrane Library from inception to November 28, 2025
Search Strategy:
#1. MeSH descriptor: [Methadone] explode all trees
#2. (Methadone):ti,ab,kw OR (Phenadone):ti,ab,kw OR (Physeptone):ti,ab,kw OR (Dolophine):ti,ab,kw OR (Amidone):ti,ab,kw
#3. (Methadone Hydrochloride):ti,ab,kw OR (Hydrochloride, Methadone):ti,ab,kw OR (Biodone):ti,ab,kw OR (intraoperative methadone):ti,ab,kw OR (methadon):ti,ab,kw
#4. #1 OR #2 OR #3

#5. MeSH descriptor: [Cardiac Surgical Procedures] explode all trees
#6. (Cardiac Surgical Procedures):ti,ab,kw OR (Heart Surgical Procedure):ti,ab,kw OR (Cardiac Surgery):ti,ab,kw OR (Heart Surgery):ti,ab,kw
#7. (heart operation):ti,ab,kw OR (myocardial resection):ti,ab,kw OR (Procedures, Cardiac Surgical):ti,ab,kw OR (surgery, heart):ti,ab,kw
#8. #5 OR #6 OR #7
#9. MeSH descriptor: [Analgesics, Opioid] explode all trees
#10. (Analgesics, Opioid):ti,ab,kw OR (narcotic analgesic agent):ti,ab,kw OR (Opioids):ti,ab,kw OR (Full Opioid Agonists):ti,ab,kw OR (Agonist-Antagonists, Opioid Mixed):ti,ab,kw
#11. (Partial Agonists, Opioid):ti,ab,kw OR (Opioid Partial Agonists):ti,ab,kw OR (Partial Opioid Agonists):ti,ab,kw
#12. #9 OR #10 OR #11
#13. #4 AND #8 AND #12
Results: 31 records

Database: MEDLINE from inception to November 28, 2025
Search Strategy:
#1. Methadone/
#2. (Phenadone or Physeptone or Phymet or Dolophine or Amidone or "Methadone Hydrochloride" or "Hydrochloride, Methadone" or Biodone or Metadol or Metasedin or Methaddict or Methadose or Methex or Pinadone or Symoron or "intraoperative methadone" or methadon).mp.
#3. #1 OR #2
#4. Cardiac Surgical Procedures/
#5. (Surgical Procedure Cardiac or Surgical Procedures Cardiac or Heart Surgical Procedure or Heart Surgical Procedures or Procedure Heart Surgical or Procedures Heart Surgical or Surgical Procedure Heart or Surgical Procedures Heart or Cardiac Surgical Procedure or Procedures Cardiac Surgical or Procedure Cardiac Surgical or Cardiac Surgery or Heart Surgery or Cardiosurgery or heart operation or myocardial resection or surgery heart).mp.
#6. #4 OR #5
#7. Analgesics, Opioid/
#8. (Analgesics Opioid or Opioid Agonists or Opioid Partial Agonists or Opioid Agonist-Antagonists or Narcotics).mp.
#9. #7 OR #8
#10. #3 AND #6 AND #9
Results: 23 records

Supplementary Table 2. Characteristics of the Included Studies

Study	Country	Study design	Sample size (Methadone/ Control)	Type of surgery	Intervention measures (Methadone/ Control)	Treatment dose (Methadone/ Control)	Follow-up duration
Wong2025	China	Randomized controlled trials	44/42	CABG,valve replacement,Combined CABG and valve surgery(Elective sternotomy)	Methadone/ Morphine	0.2 mg/kg / 0.2 mg/kg	72 h
Murphy2015	USA	Randomized controlled trials	77/79	CABG,valve replacement,Combined CABG and valve surgery(With CPB)	Methadone/Fentanyl	0.3 mg/kg / 12 μg/kg	72 h
Carvalho2018	Brazil	Randomized controlled trials	50/50	CABG(Off-pump)	Methadone /Morphine	0.1 mg/kg / 0.1 mg/kg	36 h
Udelsmann2011	Brazil	Randomized controlled trials	18/19	cardiac surgery(With CPB)	Methadone /Morphine	20mg /Not reported	24 h
LaColla2024	USA	retrospective cohort study	172/117	CABG,valve replacement,Combined CABG and valve surgery(Elective sternotomy)	Methadone/Intrathecal Morphine	0.1 mg/kg/ 0.25mg	30 d
Edwards2024	USA	retrospective cohort study	1997/4386	CABG,valve replacement,Combined CABG and valve surgery	Methadone/Hydromorphone or Fentanyl	Not reported	7 d
Wang 2022	USA	retrospective cohort study	52/65	CABG,valve replacement,Combined CABG and valve surgery(With CPB)	Methadone/Hydromorphone, Morphine or Fentanyl	0.1-0.4 mg/kg Not reported	48 h
Eisenbraun2023	USA	retrospective cohort study	775 /2307	CABG,valve replacement,Combined CABG and valve surgery(With CPB,Elective sternotomy)	Methadone /Fentanyl	Not reported	7 d

CABG,Coronary Artery Bypass Grafting; CPB, cardiopulmonary bypas; Off-pump, Off-pump coronary artery bypass grafting; h, hours; d, days.
Treatment dose:Not reported" indicates that dosing information was not available in the original publication

Supplementary Table 3. Newcastle–Ottawa Scale (NOS) scores for included observational studies

Study	Selection				Comparability	Outcome			NOS Score
	Representativeness of the exposed cohort	Selection of the non exposed cohort	Ascertainment of exposure	Demonstration that outcome of interest was not present at start of study	Comparability of cohorts / cases and controls	Assessment of outcome	Adequacy of follow-up of cohorts	Was follow-up long enough for outcomes to occur	
LaColla2024	*	*	*	*	*	*	*	*	8
Edwards2024	*	*	*	*	*	*		*	7
Wang 2022	*	*	*	*	*	*		*	7
Eisenbraun2023	*	*	*	*	*	*		*	7

Supplementary Table 4. Sensitivity Analysis of Postoperative 24-hour pain score Between Methadone Group and Control Group

Study	Z effect	SMD	95%CI	I^2	P value
Edwards2024	2.39	-0.50	-0.92 to -0.09	79%	0.02
Wang2022	3.52	-0.65	-1.01 to -0.29	77%	0.0004
Carvalho2018	2.71	-0.45	-0.78 to -0.13	82%	0.007
Murphy2015	2.63	-0.32	-0.56 to -0.08	61%	0.008
Udelsmann2011	2.78	-0.36	-0.62 to -0.11	75%	0.006
Wong2025	2.89	-0.47	-0.80 to -0.15	82%	0.004

Supplementary Table 5. GRADE summary of findings for perioperative intravenous methadone versus conventional opioids in adult cardiac surgery

Outcomes	Included studies	Participants	Mean Difference (95% CI)	I^2 (%)	*p*-value	Certainty Of Evidence (GRADE)
Postoperative pain score at 24 h	6	6832	SMD-0.44(-0.71 to 0.17)	77	0.002	Moderate ⊕⊕⊕◯
Postoperative opioid consumption within 24 h	5	3724	SMD−0.72(−1.35 to −0.23)	97	0.02	Low ⊕ ⊕◯◯
Time to first morphine rescue	4	369	MD64.46(−83.37 to 212.29)	89	0.39	Low ⊕ ⊕◯◯
Hospital length of stay	4	9871	MD0.08(-0.34 to 0.50)	89	0.71	Low ⊕ ⊕◯◯
Time of ICU discharge	6	10103	MD−4.32(−11.06 to 2.42)	97	0.21	Low ⊕ ⊕◯◯
Extubation Time	8	10203	MD,−0.06(−0.88 to 0.76)	94	0.88	Low ⊕ ⊕◯◯
Postoperative nausea	4	371	OR,0.85(0.66to 1.08)	0	0.18	Low ⊕ ⊕◯◯
Postoperative vomiting	4	371	OR,0.99(0.65to 1.50)	15	0.96	Low ⊕ ⊕◯◯
Nausea/Vomiting	3	9754	OR,0.96(0.93to 1.00)	0	0.04	Moderate ⊕⊕⊕◯
Reintubation	2	6672	OR,0.75(0.58to 0.96)	0	0.02	Low ⊕ ⊕◯◯

	Experimental			Control				Std. Mean Difference
Study or Subgroup	Mean	SD	Total	Mean	SD	Total	Weight	IV, Random, 95% CI
Carvalho2018	1.9	2.2	50	2.9	2.6	50	18.4%	-0.41 [-0.81, -0.02]
Edwards2024	1.2	1.78	1994	1.87	2.44	4352	36.3%	-0.30 [-0.35, -0.24]
Murphy2015	4.67	2.22	75	7	2.96	77	0.0%	-0.88 [-1.22, -0.55]
Udelsmann2011	0.5	0.71	18	1.84	1.38	19	8.8%	-1.18 [-1.89, -0.48]
Wang2022	3.2	1.6	52	3.1	1.2	65	20.0%	0.07 [-0.29, 0.44]
Wong2025	2.67	2.22	40	3.33	2.22	40	16.5%	-0.29 [-0.74, 0.15]
**Total (95% CI)**			**2154**			**4526**	**100.0%**	**-0.32 [-0.56, -0.08]**

Heterogeneity: $Tau^2 = 0.04$; $Chi^2 = 10.33$, df = 4 (P = 0.04); $I^2 = 61\%$
Test for overall effect: Z = 2.63 (P = 0.008)

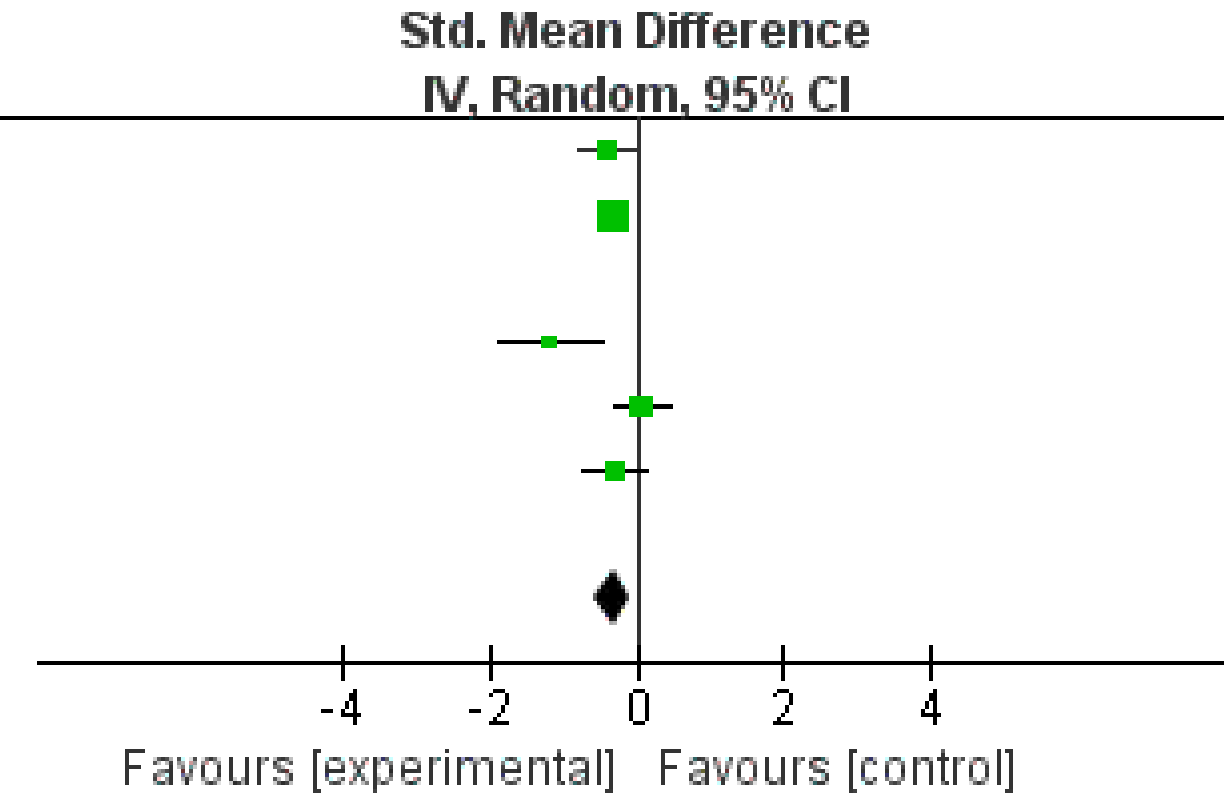

Supplementary Figure 1. Leave-One-Out Sensitivity Analysis of Postoperative 24-Hour Pain Intensity After Exclusion of Murphy et al

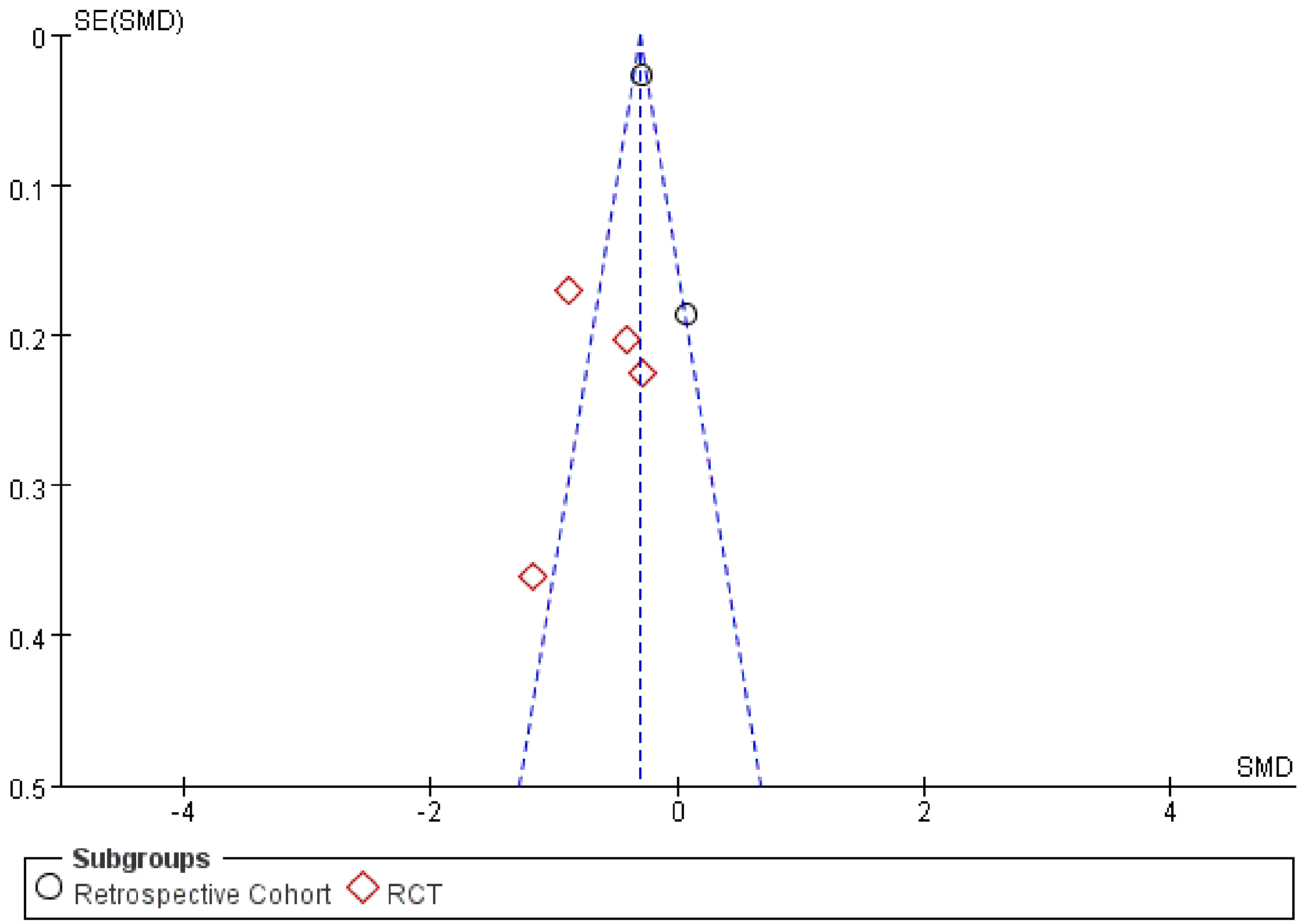

Supplementary Figure 2. Funnel plot for postoperative pain score at 24 h

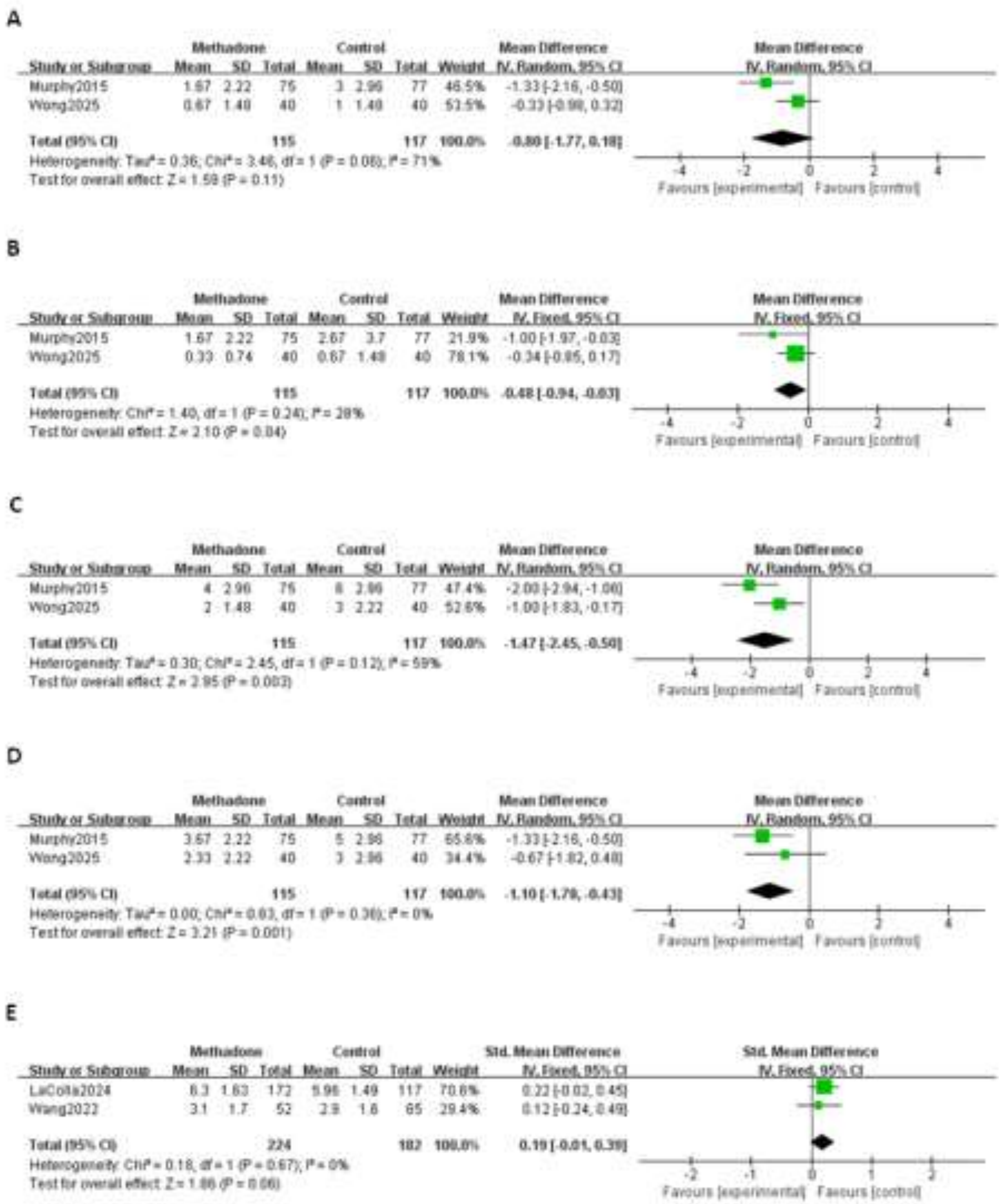

Supplementary Figure 3. Postoperative pain intensity at additional time points.
A, pain at rest at 48 hours; B, pain at rest at 72 hours; C, pain during coughing at 48 hours; D, pain during coughing at 72 hours; E, average pain intensity within 24 to 48 hours postoperatively.

Study or Subgroup	Experimental Mean	Experimental SD	Experimental Total	Control Mean	Control SD	Control Total	Weight	Std. Mean Difference IV, Random, 95% CI
Eisenbraun2023	40.83	38.89	775	183	120	2307	0.0%	-1.35 [-1.43, -1.26]
LaColla2024	23.31	14.73	172	23.96	18.8	117	27.4%	-0.04 [-0.27, 0.20]
Murphy2015	21.99	17.79	77	38.01	35.55	79	25.8%	-0.56 [-0.89, -0.24]
Wang2022	22.1	31.48	52	40.7	44.07	65	24.7%	-0.47 [-0.84, -0.10]
Wong2025	30	24.45	40	84	57.78	40	22.1%	-1.21 [-1.68, -0.73]
**Total (95% CI)**			**341**			**301**	**100.0%**	**-0.54 [-0.99, -0.09]**

Heterogeneity: Tau² = 0.17; Chi² = 21.05, df = 3 (P = 0.0001); I² = 86%
Test for overall effect: Z = 2.38 (P = 0.02)

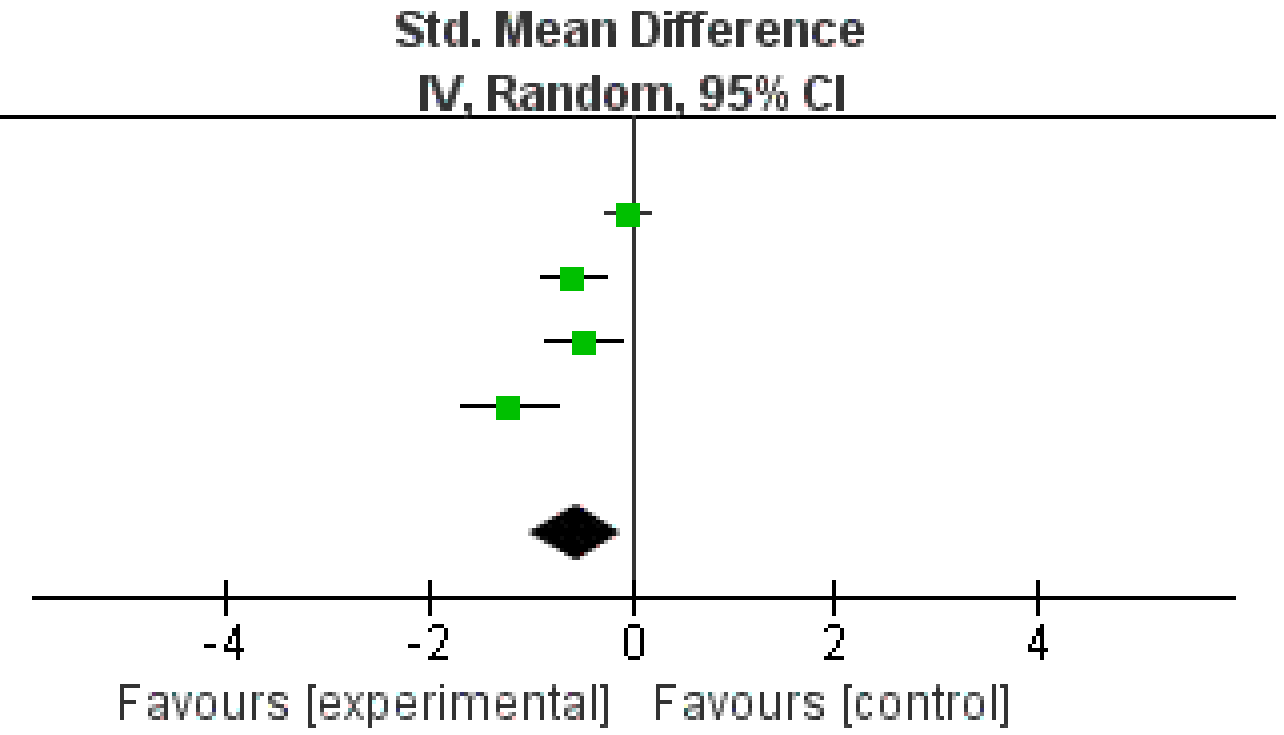

Supplementary Figure 4. Forest plot for the sensitivity analysis of postoperative 24-hour opioid consumption after exclusion of Eisenbraun et al

Study or Subgroup	Experimental Mean	Experimental SD	Experimental Total	Control Mean	Control SD	Control Total	Weight	Mean Difference IV, Random, 95% CI
Carvalho2018	145.9	178.5	50	269.4	252.9	50	0.0%	-123.50 [-209.30, -37.70]
Murphy2015	379.8	266.4	75	220.2	189	77	49.2%	159.60 [86.00, 233.20]
Udelsmann2011	855.17	593.39	18	625.53	525.23	19	2.0%	229.64 [-132.19, 591.47]
Wong2025	235	217.78	40	143.33	97.78	40	48.7%	91.67 [17.69, 165.65]
**Total (95% CI)**			**133**			**136**	**100.0%**	**127.92 [76.28, 179.57]**

Heterogeneity: Tau² = 0.00; Chi² = 1.94, df = 2 (P = 0.38); I² = 0%
Test for overall effect: Z = 4.86 (P < 0.00001)

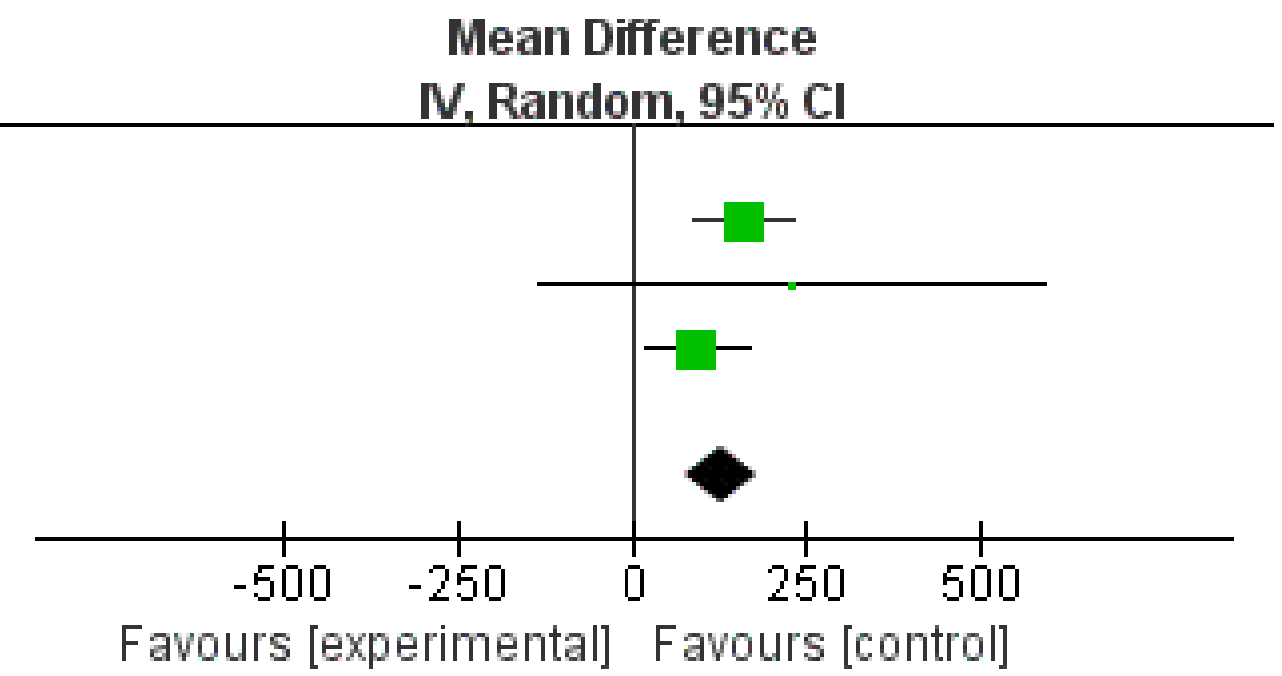

Supplementary Figure 5. Forest plot for the sensitivity analysis of time to first rescue morphine administration after exclusion of Carvalho et al

	Experimental			Control				Mean Difference
**Study or Subgroup**	**Mean**	**SD**	**Total**	**Mean**	**SD**	**Total**	**Weight**	**IV, Random, 95% CI**
Carvalho2018	4.13	3.56	50	3.78	2.55	50	10.1%	0.35 [-0.86, 1.56]
Edwards2024	5.13	3.78	1994	6.9	6.96	4352	0.0%	-1.77 [-2.04, -1.50]
Eisenbraun2023	4.33	2.37	775	4.53	2.37	2307	40.8%	-0.20 [-0.39, -0.01]
LaColla2024	5.74	5.47	172	4.51	4.47	117	11.0%	1.23 [0.08, 2.38]
Murphy2015	7	3.33	75	7.08	4.26	77	10.1%	-0.08 [-1.29, 1.13]
Udelsmann2011	7.18	5.07	18	5.98	2.99	19	2.5%	1.20 [-1.50, 3.90]
Wang2022	3.67	2.37	52	4.17	2.52	65	15.7%	-0.50 [-1.39, 0.39]
Wong2025	6.15	3.62	40	5.57	1.68	40	9.8%	0.58 [-0.66, 1.82]
**Total (95% CI)**			**1182**			**2675**	**100.0%**	**0.09 [-0.35, 0.53]**

Heterogeneity: $Tau^2 = 0.11$; $Chi^2 = 9.33$, df = 6 (P = 0.16); $I^2 = 36\%$

Test for overall effect: Z = 0.39 (P = 0.69)

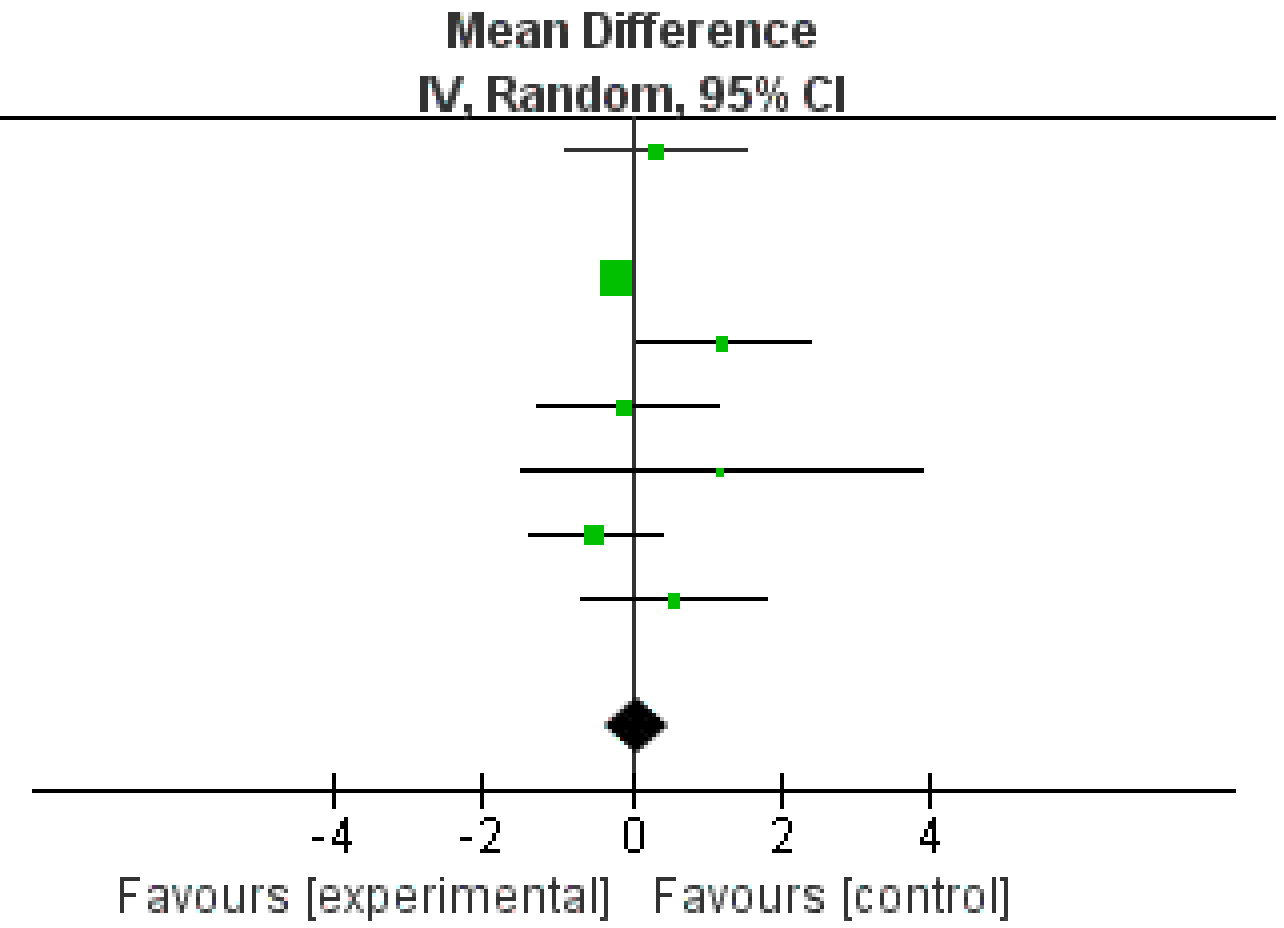

Supplementary Figure 6. Forest plot for the sensitivity analysis of time to extubation after exclusion of Edwards et al

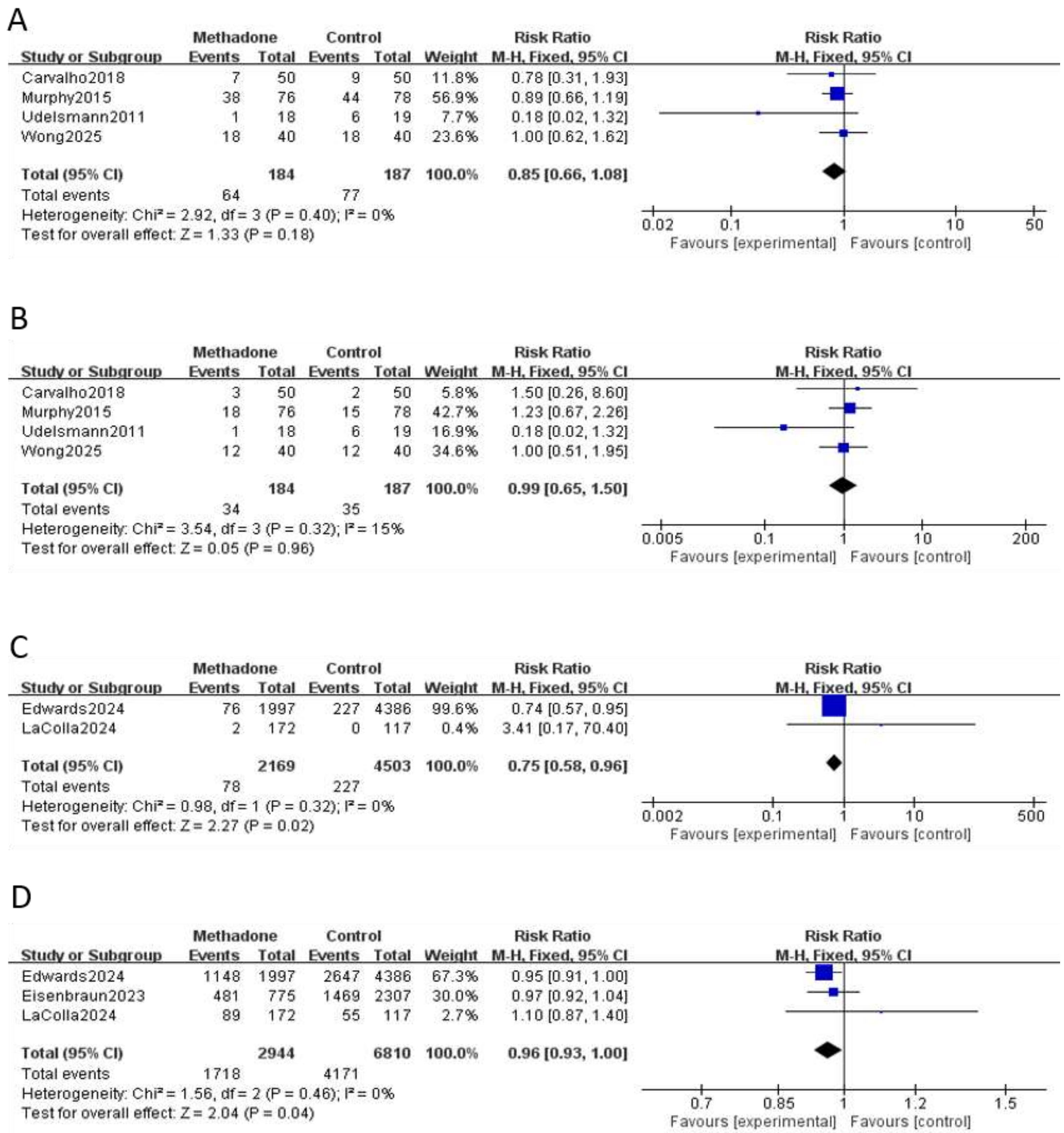

Supplementary Figure 7:Forest plots comparing postoperative outcomes between methadone and control opioids(A)Nausea; (B)Vomiting; (C) Reintubation; (D) Nausea/Vomiting.
